# Central Sleep Apnea in Adults: An Interdisciplinary Approach to Diagnosis and Management—A Narrative Review

**DOI:** 10.3390/jcm14072369

**Published:** 2025-03-29

**Authors:** Alpár Csipor Fodor, Dragoș Huțanu, Corina Eugenia Budin, Maria Beatrice Ianoși, Delia Liana Rachiș, Hédi-Katalin Sárközi, Mara Andreea Vultur, Gabriela Jimborean

**Affiliations:** 1 Pulmonology Department, County Emergency Hospital Miercurea-Ciuc, 530173 Miercurea-Ciuc, Romania; sfnkcaj@gmail.com; 2 Pulmonology Department, “George Emil Palade” University of Medicine, Pharmacy, Science and Technology of Târgu Mureș, 540139 Târgu Mureș, Romania; hutanudragos1@gmail.com (D.H.); hedi.balog@umfst.ro (H.-K.S.); mara.vultur@umfst.ro (M.A.V.); gabriela.jimborean@umfst.ro (G.J.); 3 Pathophysiology Department, “George Emil Palade” University of Medicine, Pharmacy, Science and Technology of Târgu Mureș, 540139 Târgu Mureș, Romania; corina.budin@umfst.ro; 4 Pulmonology Clinic, Mureș County Clinical Hospital, 540011 Târgu Mureș, Romania; rachis.delia-liana.23@stud.umfst.ro

**Keywords:** sleep apnea, central sleep apnea, Cheyne-stokes Apnea

## Abstract

Central sleep apnea (CSA) is a heterogeneous group of sleep-related breathing disorders characterized by intermittent absence of respiratory effort during sleep. CSA results from impaired neurological signaling from the respiratory centers to the respiratory muscles, leading to airflow cessation for at least 10 s. Major causes include heart failure, opioid use, central neurological disorders, and altitude exposure. This review outlines the pathophysiology of CSA, emphasizing ventilatory instability and brainstem dysfunction as key mechanisms. It details the classification of CSA subtypes, including Cheyne–Stokes respiration, high-altitude CSA, and drug-induced CSA. Clinical manifestations range from excessive daytime sleepiness to cardiovascular complications. Diagnostic approaches encompass polygraphy, polysomnography, and various laboratory tests to evaluate comorbidities. Treatment requires a multidisciplinary approach, addressing underlying conditions while utilizing positive airway pressure (PAP) therapy, adaptive servo-ventilation (ASV), supplemental oxygen, and pharmacological interventions. Newer modalities, such as phrenic nerve stimulation, offer promising outcomes for CSA management. This review underscores the necessity of an individualized, interdisciplinary strategy to improve patient outcomes in CSA.

## 1. Introduction

Central sleep apnea (CSA) comprises a heterogeneous group of sleep-related breathing disorders in which respiratory effort is absent intermittently during sleep. CSA is defined as the absence of airflow/very severe decrease in airflow at the nose and mouth level for at least 10 s during sleep in an adult (repeated >5 events/hour), caused by the absence of neurological impulses from the respiratory centers to the respiratory muscles; therefore, no thoracal/abdominal effort can be observed during the events [1].

The most common possible causes of CSA are systolic heart failure, opioid abuse, central neurological disorders, exposure to altitude variations, or CSA associated with severe obstructive sleep apnea (OSA). The International Classification of Sleep Disorders, Third Edition (ICSD-3rd) describes five different CSA entities that affect adults with different clinical and polysomnographic (PSG) features [2]:

- Primary CSA;
- Central Cheyne–Stokes Apnea (CC-SA);
- High-altitude CSA;
- CSA due to medical conditions other than Cheyne–Stokes;
- CSA due to drugs or other noxious substances.

## 2. Mechanisms of CSA Production and Main Causes

The mechanism of CSA production, unlike OSA (which presents respiratory effort), consists in the lack of transmission of nervous impulses from the respiratory centers to the respiratory muscles to trigger periodic breathing [2,3,4]. CSA is found in approximately 5% of all cases of sleep apnea [4]. Normal ventilation is tightly regulated to maintain arterial oxygen (PaO_2_) and carbon dioxide (PaCO_2_) levels within narrow normal ranges. This is achieved through feedback phenomena through the intervention of several components: peripheral and central chemoreceptors, intrapulmonary vagal receptors, respiratory control-centers in the brainstem, and respiratory muscles [3].

During wakefulness, signals from the cortex influence breathing through behavioral control. During sleep, behavioral control is lost, and chemical control is the major mechanism for regulating ventilation, with PaCO_2_ being the major stimulus for ventilation. CSA is most often observed during non-rapid eye movement (NREM) sleep, when behavioral influence is minimal. Sleep associates increased PaCO_2_ and a higher PaCO_2_ apnea threshold. Reductions in PaCO_2_ of only a few mmHg can lead to apnea [3,4]. Two major pathophysiological events cause CSA: (1) Ventilatory instability; (2) Depression of brainstem respiratory-centers or chemoreceptors [5,6].

Ventilatory instability is the mechanism behind CSA, CC-SA, high altitude periodic breathing, and primary CSA [3,4,5]. Ventilation is particularly at risk for instability when resting PaCO_2_ approaches the apneic threshold of PaCO_2_. Patients with hypocapnia and heart failure and those ascending to high altitudes often develop these conditions, with predisposition to a periodic breathing pattern. Hypoxia increases the ventilatory response to changes in PaCO_2_ and predisposes to ventilatory instability. During wakefulness, breathing is regulated by both behavioral and chemical control mechanisms, but upon sleep onset, behavioral control is lost, making ventilation entirely dependent on chemical feedback. This transition increases the apneic threshold, meaning that smaller drops in PaCO_2_ can suppress respiratory drive, promoting sleep-onset central apneas. In conditions like heart failure and high altitude, CO_2_ regulation is further altered. Heart failure leads to chronic hyperventilation and hypocapnia due to increased chemoreceptor sensitivity, predisposing patients to Cheyne–Stokes respiration and central sleep apnea. Similarly, at high altitude, hypoxia-driven hyperventilation lowers PaCO_2_, shifting it closer to the apneic threshold, increasing the risk of periodic breathing. These conditions also elevate loop gain, a measure of ventilatory control instability, where heightened sensitivity to CO_2_ fluctuations results in exaggerated ventilatory responses, making individuals more prone to central apnea events [6,7,8].

CSA-hypoventilation syndromes, such as those associated with narcotic use or brainstem lesions, are due to disturbances in the respiratory centers and/or peripheral chemoreceptors that may become more apparent during sleep due to the suppression of wakefulness.

Narcotics (heroin, morphine, and methadone) cause respiratory depression by stimulating opioid receptors on neurons located in the respiratory centers of the medulla. Although tolerance to many of the central nervous system effects of opioids develops, studies [9,10] have demonstrated abnormal hypercapnic and hypoxic ventilatory responses in chronic narcotic users. CSA is common especially in individuals who use opioids long-term [11,12].

Sometimes the mechanisms responsible for CSA and OSA overlap, and patients with CSA often present obstructive events. Studies have shown that the hypopharynx can be significantly narrowed during a CSA event. During normal inspiration, neuronal stimulation occurs in the diaphragm and upper airway muscles (UAMs) that stiffen and dilate the pharynx to keep it open. If there is a decrease in activity in both the diaphragm and the UAM dilators, the result could be CSA or OSA. If the UAMs remain open, the event will be CSA. If the UAMs are closed during CSA and diaphragmatic activity resumes before pharyngeal dilator muscle tone is restored, a mixed apnea results [12]. Thus, susceptibility to UAM collapse may determine whether OSA occurs due to ventilatory instability.

CSA is more common in the elderly than in the middle-aged, and it is quite rare compared to OSA (about 2–3% of all sleep apnea) [5,9]. CSA is prevalent in men and less common in postmenopausal women. The explanation for this discrepancy is the presence of a lower apneic threshold of PaCO_2_ in women compared to men. Thus, women require a greater reduction in PaCO_2_ to initiate apnea than men [8].

Medical causes (associated with CC-SA or CSA) [13,14,15] (Box 1).

- Heart failure, severe arrhythmias;
- Renal failure;
- Stroke, brain tumors, encephalitis, Parkinson’s disease, lesions of the medullary centers by neoplasms, infarctions, infections;
- “Shy Drager” syndrome with orthostatic hypotension and extrapyramidal syndrome;
- Post-poliomyelitis syndrome (progressive muscle weakness);
- Muscular dystrophy, myasthenia gravis.
- Diabetes mellitus;
- Hypothyroidism; acromegaly;
- “Arnold Chiari” syndrome types I–III (congenital condition of the cerebellum that herniates into a neighboring anatomical compartment);
- “Prader Willi” syndrome (genetic disease with muscle hypotonia, slowed development and growth, obsessive–compulsive behavior, hyperphagia, and obesity).

Box 1 **ICSD 3 Criteria for Cheyne–Stokes respiration [1,2].**

A. Presence of one or more symptoms—drowsiness, difficulty maintaining or inducing sleep, frequent awakenings and restless sleep, awakening due to shortness of breath;

B. Association with atrial fibrillation/flutter, heart failure, neurological disorder (stroke/tumors);

C. PSG polysomnography (during diagnosis or CPAP titration) shows the following:

             1. ≥5 central apneas and hypopneas/hour of sleep.

             2. The total number of CSA and/or CSH is >50% of the total number of apneas and hypopneas.

           3. The ventilatory “pattern” meets the criteria for CC-SA (at least a series of 3 consecutive CSA/CSH separated by periods of crescendo–decrescendo breathing with a cycle length from the onset of the apnea to the onset of the next of ≥40 s).

D. The illness cannot be better explained by another sleep disorder, medical or neurological disorder, or the use of a medication or substance.

High-altitude CSA can occur above ≥5000 m altitude. The breathing patterns are Cheyne–Stokes type with a shorter cycle of apnea and hyperventilation (15–35 s) [1,3,4].

Use of opioids and other central nervous system depressants [10,11] is easily diagnosed by history. Of the participants in a stable methadone maintenance program, 30% had a CSA index >5 and 20% >10. Methadone blood concentration was significantly associated with CSA severity.

Primary (idiopathic) CSA has an unknown etiology characterized by CSA without CC-SA pattern [10,16,17]. It can be described when ≥5 CSA occur, each >10 s/h of sleep. PaCO_2_ is normal or low. CSA ends abruptly with a large breath and without associated hypoxemia. It is rare (one study highlights 3.8% of 650 CSA diagnosed by PSG) [18]. In the history of the sleep laboratory of the Pulmonology Clinic of Targu Mures, the cases were severe and were associated with cognitive decline/dementia and arrhythmias without being able to establish a link between them as triggering factors or as consequences of CSA.

**Figure 1:**
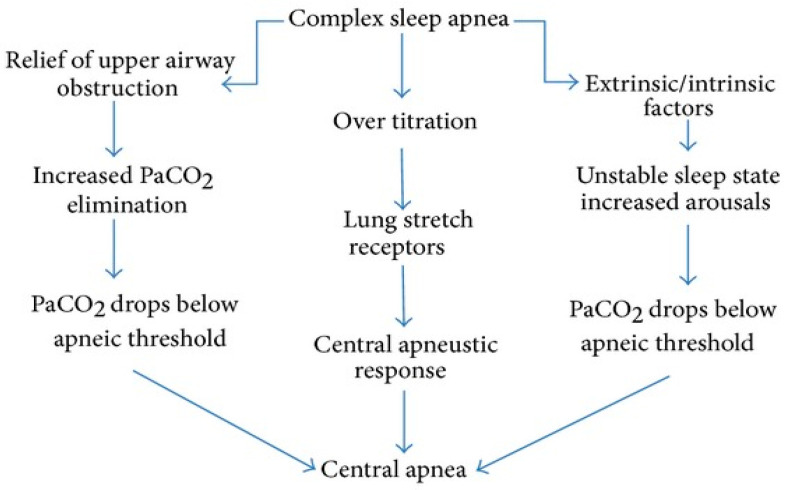
Complex sleep apnea syndrome/emergent CSA detection algorithm [1,15].

CSA associated with titration or CPAP therapy (emergent CSA, previously referred to as “complex sleep apnea”) may occur during the first CPAP titration for primary OSA (6.5%), and it is mainly due to ventilatory instability caused by CPAP’s effect on carbon dioxide regulation. Only 1.5% of patients develop persistent CSA associated with long-term persistent CPAP therapy. Key pathways leading to central apnea in complex sleep apnea are characterized by relief of upper airway obstruction, when CPAP therapy eliminates airway obstruction, leading to increased ventilation causing excess CO_2_ elimination, lowering arterial PaCO_2_. If PaCO_2_ drops below the apneic threshold, the brainstem’s respiratory drive is inhibited, resulting in central apnea. Another causing mechanism can be the over-titration of CPAP pressure, when excessively high CPAP pressure can overstimulate lung stretch receptors, which triggers a central apneustic response, possibly leading to central apneas. Furthermore, factors such as sleep fragmentation, arousals, and underlying ventilatory instability can further contribute to CO_2_ fluctuations. An unstable sleep state with frequent arousals may increase the likelihood of PaCO_2_ dropping below the apneic threshold, leading to central apnea (Figure 1) [1,15].

Most apneas resolve after a few weeks of proper treatment of OSA [15]. CSA also occurs in healthy individuals during the wake–sleep transition, when chemoreceptors reset and instability of ventilatory control occurs, with the events being brief and not associating with significant O_2_ desaturation. Once the wake–sleep transition is completed, normal individuals should not present more than 5 events/h of sleep during the night [15,19,20].

Common risk factors for CSA are frequently encountered as complex forms of heart failure due to systolic deficit, significant rhythm disturbances, cerebrovascular and central nervous diseases in general, and in case of opioid use [19].

## 3. Symptoms of Central Sleep Apnea

CSA presents daytime and nocturnal symptoms, but also evident etiological factors and complications, such as the following [4]:

- Excessive daytime sleepiness (less than OSA); lack of energy and daytime fatigue;
- Insomnia or awakenings—restless sleep;
- Apnea reported by sleep partners;
- Paroxysmal nocturnal dyspnea;
- Signs of underlying causes with frequent association with the following:
  - Cardiovascular disorders (atrial fibrillation, flutter, ventricular ectopy, other nocturnal arrhythmias, heart failure) [14,15,16,21];
  - Neurological diseases (stroke, tumors, Parkinson’s disease, encephalitis);
  - Neuromuscular disorders, muscular dystrophy, myasthenia gravis;
  - Chronic kidney disease;
  - Living at high altitude or recent variations in altitude;
  - Drug abuse (methadone, opioids);
  - Presence of obesity (BMI > 30 kg/m^2^) more strongly correlates with OSA rather than CSA.

## 4. Specific Laboratory Investigations in Sleep Apnea

**Figure 2:**
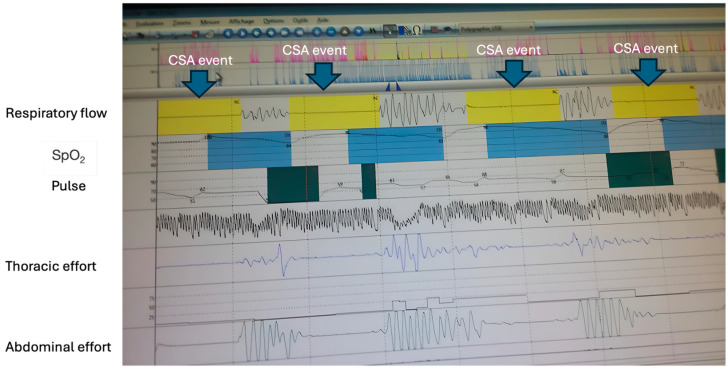
Central sleep apnea (polygraphy—Pulmonology Clinic Mureș). Absence of airflow, absence of respiratory movements of the chest and abdomen. CSA—central sleep apnea; SpO_2_—blood oxygen saturation.

Sleep apnea should be investigated in patients with the help of the following:

- Questionnaires for suspicion of daytime sleepiness (Epworth) and sleep apnea risk (Stop Bang Questionnaire, Berlin) should be applied to all patients with sleep apnea suspicion, before correctly differentiating OSA from CSA through more complex sleep study investigations;
- Pulse oximetry; blood gas analysis;
- Polygraphy/home sleep testing/level 3 testing (PG) and polysomnography/level 1 testing (PSG) with the following findings:
  (a) Central sleep apnea CSA (Figure 2) [1,2,3,4]:
  - Lack of airflow at nose and mouth level for >10 s during sleep in an adult;
  - Lack of respiratory effort of the thoracic and abdominal muscles;
  - Nocturnal micro-awakenings;
  - Hypoxemia +/− hypercapnia.
  (b) Central sleep hypopnea CSH [1,2,3,4]:
  - Decrease in airflow at the nose or mouth over 30% of the pre-event value, ≥10 s, together with absence of inspiratory effort and desaturation of +3% with or without arousal associated to the event; this is important to report, while frequently difficult to identify, due to multiple factors overlapping, such as the presence of insufficient respiratory effort or even lack in sensitivity of monitoring devices.
  (c) Cheyne–Stokes respiration/Central Cheyne–Stokes Apnea (CC-SA) (periodic ventilation) is characterized by respiratory events during sleep (succession of at least 3 events), with a progressive periodic amplification of ventilation “crescendo/decrescendo pattern” and then, with its progressive decrease, alternating with CSA/CSH [1,2]. The duration of the respiratory cycle from the onset of ventilation to the onset of another ventilation is ≥40 s [1].
  (d) Mixed apnea (MSA) is characterized by apnea during sleep that begins as a CSA (without thoraco-abdominal effort) and is followed by an OSA (ventilatory movements of the chest and abdomen).
- Associated tests for causes and comorbidities: serological tests—glycemia, glycosylated Hb, LDL, HDL, uric acid, anti-acetylcholine receptor antibodies, blood gas analysis, renal function tests, ENT examination, EKG, cardiological examination, cardiac ultrasound, coronary angiography, spirometry, COPD Assessment test (CAT), 6-min-walk-test, imaging tests (MRI, brain CT), neurological and endocrinological examination, capnography.
- Blood gas analysis may reveal respiratory alkalosis (PaCO_2_ < 40 mm Hg while awake) in patients with primary CSA, high-altitude periodic breathing, and CC-SA.

### 4.1. Cardio-Respiratory Ventilatory Polygraphy (PG)

PG evaluates several parameters (through 4–6 channels) during sleep [1,22,23,24]: nasal/oral airflow; respiratory effort (respiratory movements of the chest and abdomen); SaO_2_; patient position during sleep; and pulse and pulse variability.

Advantages of PG: PG is accepted in the diagnosis of uncomplicated sleep apnea in which the clinical examination is suggestive [2]; it is portable and has a high accessibility; it is relatively inexpensive. PG can benefit from additional channels (EKG, video camera, body position and snoring sensors, EEG channels). The reading and validation of sleep curves is performed in a short time compared to PSG (30 min). Disadvantages of PG could be that it does not measure sleep stages, it cannot assess the sleep architecture, and it cannot detect micro-awakenings or limb movements. The exact moment when patients fall asleep and exact duration of sleep cannot be accurately determined and neither can patient behavior during sleep be investigated through PG [1,2,22,23,24].

### 4.2. Polysomnography (PSG)

Polysomnography is considered to be the reference/gold standard sleep study tool, by allowing the diagnosis of complex sleep disorders and breathing disorders during sleep, providing comprehensive data on sleep architecture, arousals, and respiratory events. It is performed in dedicated centers (sleep laboratories) where medical teams with high expertise collaborate. PSG associates complex equipment for recording different parameters during sleep [1,2,23,25]. Parameters monitored with PSG are the following:

1. Sleep structure: electrical brain activity (electroencephalography—EEG) that identifies sleep stages, microarousals; electrooculogram (EOG); chin electromyogram (EMG); anterior tibial electromyogram that can prove limb movement or atonia during sleep.
2. Respiratory events—detection of nose/mouth air flow and another sensor for snoring detection.
3. Thoracal and abdominal movements detected by 2 strips attached to the patient.
4. Repercussions of respiratory events: (a) Consequences on sleep structure (sleep fragmentation); (b) Gasometry (nocturnal SaO_2_ and PaCO_2_) and cardiovascular repercussions through EKG; pulse measurement should be considered when identifying the potential mechanism of CSA.
5. Patient behavior recorded by PSG-associated camera and by a position sensor (“positional sleep apnea”, actigraphy).

PSG allows the diagnosis of various sleep disorders: central apneas, narcolepsy, parasomnias, complex apneas, behavioral disorders during REM sleep, aspects related to legal medicine, periodic limb movements, restless legs syndrome, insomnia, circadian rhythm disorders, and hypoxia of various causes [23,25].

CSA is more common in stages 1 and 2 of sleep, and awakenings are frequent, which fragments sleep and prevents patients from entering into deep delta-sleep. Events are less often encountered in REM. The duration of an apnea/ventilation cycle is <45 s [10]. In the CC-SA cycle in heart failure, CSA is determined by awakening and increased tidal volume associated with decreased PaCO_2_. As the patient falls asleep, the apnea threshold also rises, and the PaO_2_ and ventilation oscillates around this threshold. The duration of a ventilatory cycle (apnea–hyperpnea) is ≥45 s and depends on cardiac output [2]. Altitude CSA occurs in NREM sleep and improves within a few days with altitude adaptation. Substance-induced CSA occurs in NREM sleep [11].

## 5. Assessment of Comorbidities

1. Investigation of the cardiovascular system:
  - EKG, cardiorespiratory exercise test, blood pressure, pulse measurement;
  - Cardiac ultrasound (often the ejection fraction is <40%);
  - Imaging investigations—ultrasound, magnetic resonance imaging, positron emission tomography of myocardial perfusion;
  - Coronary angiography;
2. Investigation of metabolic syndrome (BMI, waist circumference), blood count, blood sugar, glycosylated Hb, expanded lipidogram;
3. Neuropsychiatric consultation.

## 6. Treatment of Central Apnea

The treatment of CSA should be managed by a multidisciplinary team—pulmonologist, cardiologist, physiotherapist, and specialist of the internal medicine (to treat underlying cause of CSA) when secondary causes of CSA might be present and treatment and recommendations from a sleep specialist could overlook the presence of significant comorbidities. The main objectives are to stabilize sleep and treat the causes that led to CSA. The treatment of CSA is more difficult than the treatment of OSA and will be personalized for each patient.

Positive airway pressure (PAP) devices are the standard treatment and are initiated alongside treatment of the underlying cause of CSA. Treatment of underlying conditions—heart failure, renal failure, OSA, descent to lower altitudes (in those living at higher altitudes), tapering of opioid medications—is important (the dose may be reduced). Exercise has no direct effect on CSA linked to heart failure [26]. Other useful treatments include oxygen therapy and some pharmacological agents.

### 6.1. Treatment with Positive Airway Pressure (PAP)

Devices can be initiated as CPAP—continuous PAP, bilevel PAP therapy, and adaptive servoventilation (ASV). Studies have shown that CPAP and BPAP are more effective for the management of CSA in heart failure and opioid use [27,28].

(a) Continuous positive airway pressure (CPAP) devices

CPAP is widely used to treat OSA: the device used during sleep delivers continuous air under pressure into the airway via a mask to keep the upper airways (UAW) open. CPAP can prevent the closure of UAW, which can trigger CSA. Several types of masks are available to ameliorate patient comfort. The air pressure can be adjusted considering the needs of each patient.

CPAP devices should be initiated, firstly in patients where CSA and OSA coexist, with positive pressure treating the obstructive component of sleep apnea and therefore stabilizing ventilation, which can reduce the central events. Furthermore, when dealing with patients with CSA due to heart failure, CPAP reduces nocturnal hypoxia, reduces left ventricular afterload, and stabilizes ventilation, therefore stabilizing PCO_2_ fluctuations that can trigger central events. However, studies show that overall survival was not improved significantly in these patients, as proved in the CANPAP trial [29].

A reference study showed that CPAP improves symptoms of CSA, cardiac performance, heart transplant-related mortality, and quality of life [27]. Other studies show that CPAP in patients with congestive heart failure was associated with improvement in CSA, improvement in nocturnal oxygenation and ejection fraction, and improvement in 6-min-walk-test distance [28,29,30,31,32,33,34].

The increase in dead space can be done by attaching a 400–800 mL piece to the mask that increases the PaCO_2_ in the inspired air and the expiratory rebreathing space, and raises the PaCO_2_ threshold for a stable ventilation (a threshold that is too low could cause CSA) [34].

(b) Bilevel positive airway pressure (BPAP)

Devices may be an alternative for those who cannot tolerate CPAP (and who might need very high CPAP pressures that interfere with expiration) or who do not respond to CPAP. Bilevel PAP devices create a positive pressure difference (between IPAP and EPAP) and are recommended especially for patients with hypercapnic CSA with hypoventilation and for those who require high PAP values. Also, some patients with CC-SA, heart failure or primary CSA may benefit from BPAP [32], as well as those with CSA related to opioid use [33].

BPAP devices are set with inspiratory positive airway pressure levels (IPAP) and expiratory positive airway pressure levels (EPAP).

IPAP provides ventilatory support during inspiration (increases alveolar volume), in the following ways:

- Decreases respiratory effort (takes over the effort of the inspiratory muscles);
- Increases tidal volume and ventilation/minute;
- Improves gas exchange;
- Decreases the apnea–hypopnea index.

EPAP is the pressure provided by the ventilator while the patient exhales.

- Keeps the upper airways (UAW) open during sleep in those with unstable CC-SA;
- Reduces the mechanical work and respiratory effort due to end-expiratory pressure;
- Recruits alveoli and improves oxygenation;
- Eliminates obstructive apneas.

Back-up respiratory frequency (BRF) is useful in long apneas, ensuring a desired respiratory frequency if the patient has no trigger for a period of time, or when he is in apnea as well as when the patient’s inspiration effort is not effective [35]. The bed will be inclined at 45–60° (in those who require high pressures), and all devices should benefit from a humidifier and personalized masks (preferred oronasal) that lead to an increase in comfort, and thus in patient compliance [36,37].

(c) Adaptive servo ventilation (ASV)

The ASV device provides individualized ventilatory support (IPAP and EPAP) based on the detection of apneas (IPAP is increased compared to EPAP ensuring ventilatory support). The ASV principle is based on continuous monitoring of patients’ breathing patterns, together with dynamic pressure titration [37]. It is recommended for patients with respiratory failure (especially with CC-SA or opioid use) but with preserved cardiac ejection fraction (EFLV). Several studies have shown that in patients with low EFLV (NYHA 2–4, EFLV ≤ 45), ASV may increase mortality [38,39]; however, more recent research has shown that ASV does not impact all-cause mortality in these patients [40]. However, observing this controversy, the recommendation of ASV in patients with symptomatic heart failure should be considered by balancing the pros and cons, inside a multidisciplinary team, in order to avoid all risks. ASV has been effective and superior to CPAP in patients with preserved EF. ASV detects CSA, and provides an IPAP that compensates the lack of nerve impulses to the muscles and a back-up respiratory rate, improving AHI, EFLV, symptoms, and sleep architecture and decreasing daytime sleepiness [41,42]. ASV decreases heart rate and heart rate variability as well as CC-SA. ASV has been proven to decrease brain-natriuretic peptides in people with heart failure [43]. Monitoring of the effects of ASV by clinical exam, PSG, and cardiac ultrasound is necessary.

### 6.2. Other Treatment Recommendations

Nocturnal supplemental oxygen is beneficial in patients with heart failure by increasing EFLV, reducing hypoxemia and hyperventilation secondary to changes in PaCO_2_, minimizing the respiratory effort, and overall improving quality of life [37,44]. Oxygen is also beneficial in CSA caused by altitude factors, by allowing patients to maintain normal levels of PO_2_ and PCO_2_ when in high altitude locations.

Drugs that can improve CSA include acetazolamide, theophylline, and some hypnotic agents. Unfortunately, they do not have approval for common use in CSA and remain investigational drugs [45].

Acetazolamide is a carbonic anhydrase inhibitor that causes metabolic acidosis and increases basal ventilation. It would be useful (although is not indicated prophylactically) in primary CSA and in CC-SA caused by heart failure and altitude. It allows acclimatization to reduce the risk of cerebral edema and altitude sickness [45]. Some hypnotics (temazepam, zolpidem) can reduce wakefulness, unstable sleep, and arousals, especially in the period of sleep–wake transition [46]. Zolpidem was useful both in initiating sleep and in maintaining it. It reduced CSA and AHI without producing or worsening OSA [45]. Mirtazepam and Buspirone seem to have encouraging results [47,48] in reducing hypocapnia in people with spinal cord injuries. Theophylline is useful in CSA due to heart failure and altitude-induced CSA [49].

Some invasive procedures have been shown to reduce CSA and obstructive CSA (overdrive atrial pacing in patients with pacemakers for bradycardia) [50]. A newer therapy for CSA is electrical stimulation of the phrenic nerve. An FDA-approved device from the USA delivers an electrical impulse to the phrenic nerve for diaphragm contractions. The device is implanted under the skin, unilaterally; it is battery-powered and has been shown to reduce AHI by over 50%, decrease nocturnal awakenings, improve symptoms at 1 year (in 45%), decrease daytime sleepiness (in 44%), fatigue, and snoring (in 60% of cases), and improve overall sleep and quality of life [51,52].

### 6.3. Treatment Algorithms

When engaging in treatment strategies, the underlying cause for CSA syndrome should be taken in consideration, as follows [28,29,30,32,33,34,35,41,42,43,44,45]:

- For Primary CSA, the recommended approach is to trial CPAP first; if CSA worsens, adaptive servo-ventilation (ASV) should be considered, and if ASV fails, oxygen therapy or acetazolamide may be used.
- In Heart Failure-Related CSA, the priority is optimizing heart failure therapy; if CSA persists, ASV is recommended for patients with a left ventricular ejection fraction (LVEF) above 45%, while oxygen therapy or phrenic nerve stimulation may be considered if ASV fails.
- Emergent CSA from CPAP therapy should be monitored for 8–12 weeks; if it persists, reducing CPAP pressure or switching to BiPAP is advised, followed by ASV if necessary, and, in refractory cases, oxygen therapy or acetazolamide may be introduced.
- Opioid-Induced CSA should be managed primarily by reducing opioid dosage; if CSA persists, CPAP or BiPAP can be used, escalating to ASV if needed, with acetazolamide as an alternative if ASV fails.
- High-Altitude CSA is best addressed with acetazolamide as first-line treatment, followed by oxygen therapy if CSA persists, and CPAP or BiPAP for patients with ongoing symptoms.

## 7. Conclusions

Central sleep apnea (CSA) is a complex sleep-related breathing disorder with multiple underlying causes, including heart failure, opioid use, neurological conditions, and altitude exposure. The pathophysiology primarily involves ventilatory instability and brainstem dysfunction, leading to intermittent cessation of respiratory effort during sleep. Diagnosis relies on polysomnography and polygraphy, with treatment requiring a multidisciplinary approach. While positive airway pressure (PAP) therapy remains the standard treatment, newer modalities such as adaptive servo-ventilation (ASV), oxygen therapy, pharmacological agents, and phrenic nerve stimulation offer additional therapeutic options. Given the heterogeneity of CSA, individualized management strategies are crucial to optimizing patient outcomes and improving quality of life. Further research is needed to refine treatment protocols and explore novel interventions for CSA.
